# High-Quantum-Yield [Eu(Phen)_2_(NO_3_)_3_] Phosphor Exhibiting Zero Thermal Quenching under Real Operating Conditions (30–150 °C)

**DOI:** 10.1021/acsomega.5c13077

**Published:** 2026-02-18

**Authors:** Karla Scanda Raymundo Silva, Christian Javier Salas Juárez, Raúl Erick Guzmán Silva, Ismael Arturo Garduño Wilches, Hiram Isaac Beltrán Conde, José Guzmán Mendoza

**Affiliations:** † Centro de Investigación en Ciencia Aplicada y Tecnología Avanzada, del Instituto Politécnico Nacional. Av. Legaria 694, Col. Irrigación, Miguel Hidalgo, 11500 Ciudad de México, México; ‡ Área de Química, Departamento de Ciencias Básicas, Universidad Autónoma Metropolitana Azcapotzalco. Av. San Pablo No. 420, Nueva el Rosario, Azcapotzalco, 02128 Ciudad de México, México; § Área de Química de Materiales, Departamento de Ciencias Básicas, 27787 Universidad Autónoma Metropolitana Azcapotzalco, Av. San Pablo No. 420, Nueva el Rosario, Azcapotzalco, 02128 Ciudad de México, México

## Abstract

In this study, we present the first investigation of the temperature-dependent luminescence properties of the [Eu­(Phen)_2_(NO_3_)_3_] complex across the 30–150 °C range. The coordination environment, thermal stability, and structural analysis of the complex were studied by Fourier-transform infrared spectroscopy, thermogravimetric analysis, and powder X-ray diffraction techniques. Photoluminescence studies under excitation at 350 and 396 nm revealed the characteristic Eu^3+^ emission arising from the ^5^D_0_ → ^7^F_*J*_ (*J* = 1–4) transitions. Additionally, the complex exhibits a long luminescence lifetime (τ = 1.096 ± 0.001 ms) and a high photoluminescence quantum yield (Φ = 96%) with λ_exc_ = 350 nm. The photometric analysis confirms deep-red emission with 99% color purity and CIE 1931 chromaticity coordinates (*x*, *y*) = (0.66, 0.33). Notably, the [Eu­(Phen)_2_(NO_3_)_3_] complex exhibits zero thermal quenching over the temperature range, which could be attributed to the effective suppression of thermally activated back-energy transfer. These results demonstrate exceptional thermal robustness, highlighting its potential for thermally stable red-emitting optical applications.

## Graphical abstract

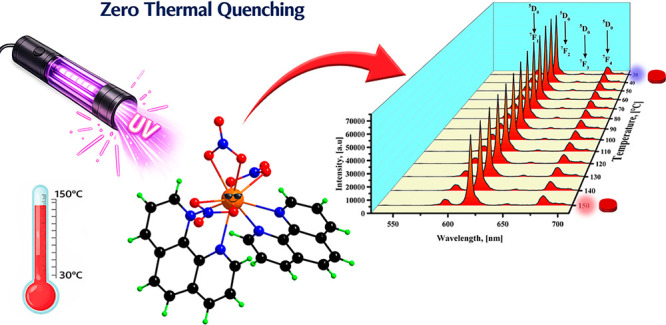

## 1 Introduction

Temperature-dependent luminescence plays a crucial role in evaluating the performance of phosphors in lighting, display, and optoelectronic applications.^1^ Most luminescent materials generally exhibit thermal quenching, in which emission intensity decreases as temperature increases as a result of enhanced nonradiative deactivation processes.^2^ Consequently, investigating the thermal stability of luminescent materials is essential for developing high-performance optical materials.

Compounds containing lanthanide ions are attractive for phosphor design due to their sharp emission lines associated with *4f–4f* transitions, long-lifetime excited states, and sensitivity to environmental changes.^3^ However, lanthanide ions are known for their low molar absorptivity and low quantum yield, limiting their emission efficiency. To circumvent these limitations, lanthanide ions have been coupled with organic ligands to form lanthanide complexes, thus enabling energy transfer mechanisms. This strategy is known as the antenna effect, which emerged as an effective path for enhancing emission intensity and quantum yield, otherwise inherently limited in bare lanthanide ions.^4^

Red phosphors play a crucial role in various applications, particularly in lighting, displays, and optical devices. Their ability to emit a deep red color is crucial for achieving high-quality color rendering in solid-state lighting systems, including light-emitting diodes (LEDs)^5^ and organic light-emitting diodes (OLEDs).^6,7^ Moreover, red phosphors contribute to color mixing in full-spectrum displays, ensuring accurate color reproduction. These materials are also utilized in sensors and medical devices, where red-emitting luminescent materials enable precise measurements and treatments,^8^ environmental monitoring,^9^ and various biological applications.^10^ Developing efficient and stable red phosphors with high quantum yield and thermal-stable luminescence is fundamental to advancing optoelectronic technologies.

Europium most commonly exists as the trivalent ion (Eu^3+^) and is widely investigated for its characteristic red photoluminescence, particularly in crystalline hosts, glasses, and coordination compounds with organic ligands. Upon excitation, emission arises from the lowest excited state, the ^5^D_0_ level (17,227 cm^–1^), to the ^7^F_*J*_ (*J* = 0–6) manifold, producing sharp, well-defined lines. Among these, the magnetic dipole ^5^D_0_ → ^7^F_1_ transition is largely insensitive to the local environment and dominates at centrosymmetric sites, whereas the hypersensitive electric dipole ^5^D_0_ → ^7^F_2_ transition becomes intense in noncentrosymmetric environments due to the relaxation of parity selection rules. Consequently, the intensity ratio *R* = *I* (^5^D_0_ → ^7^F_2_)/*I* (^5^D_0_ → ^7^F_1_) is commonly used to probe site asymmetry.^11^

Red-emitting phosphors commonly reported in the literature often have intrinsic drawbacks, such as relatively low photoluminescence quantum yields and significant thermal quenching as the operating temperature increases. For instance, conventional inorganic red phosphors like Y_2_O_3_:Eu^3+^ show a pronounced decrease in emission intensity at elevated temperatures due to the activation of nonradiative relaxation pathways, which limits their performance under real working conditions.^12^ In contrast, lanthanide-based coordination compounds, particularly those containing Eu^3+^ ions, offer an attractive alternative, as appropriate organic ligand selection enables the design of highly luminescent phosphors with improved thermal stability. The efficiency and temperature robustness of these systems could depend on the energetic alignment between the triplet ligand state and the excited levels of Eu^3+^.^13^ When an optimal energy gap is achieved between triplet-lanthanide, efficient ligand-to-metal energy transfer occurs while suppressing nonradiative processes, such as back energy transfer (BET), which are typically responsible for thermal quenching.^14^ Therefore, the rational selection of organic ligands with suitable triplet energy levels enables the development of Eu^3+^-based phosphors that combine high quantum efficiency with stable luminescent emissions as temperature increases.

In contrast, lanthanide complexes, particularly those incorporating Eu^3+^, represent a novel class of compounds distinguished by their remarkable luminescence and superior color purity. Nevertheless, these compounds tend to degrade at temperatures exceeding 400 °C. Despite this limitation, thermal stability of up to 150 °C is generally sufficient for applications involving temperature-dependent luminescence, as this represents the maximum temperature typically required in optoelectronic devices.^15^

Interest in the [Eu­(Phen)_2_(NO_3_)_3_] complex was first sparked in 1996 when Fan and Yang calculated its coordination sphere and provided a theoretical interpretation of its fluorescence spectrum,^16^ offering an initial insight into its potential optical properties. The formal structure of this complex was later reported by Sadikov et al. in 2004; however, the original study did not emphasize its luminescent properties.^17^ Over the years, further investigations have provided a more comprehensive characterization of the lanthanide complex. For instance, in 2009, Scotognella et al. successfully synthesized the [Eu­(Phen)_2_(NO_3_)_3_] complex. The authors performed an in-depth analysis, which included energy transition calculations and presented an energy-level diagram. Their work also involved a Judd-Ofelt analysis of the experimental data, as well as the measurement of the emission spectrum, an absolute quantum yield of ∼33%, and a photoluminescence lifetime of 3.5 ms for the (^5^D_0_ → ^7^F_2_) transition at room temperature.^18^ Recently, in 2018, Wang et al. conducted a comprehensive study of the photophysical processes, from UV absorption to visible emission, using theoretical calculations to further elucidate the luminescent properties of the complex.^19^ Surprisingly, to the best of our knowledge, the temperature-dependent luminescence behavior of the [Eu­(Phen)_2_(NO_3_)_3_] complex has not been extensively explored, likely due to its presumed lower degradation temperature. Nevertheless, the [Eu­(Phen)_2_(NO_3_)_3_] complex exhibits considerable thermal stability up to 150 °C, with degradation occurring near 400 °C, making it a promising candidate for applications in thermally stable luminescent materials.^20^

Moreover, also motivated by the interesting spectroscopic features of the [Eu­(Phen)_2_(NO_3_)_3_] complex, this study reports the first research on the luminescent thermal stability of the [Eu­(Phen)_2_(NO_3_)_3_] complex obtained using a simple precipitation method. For this purpose, structural and physicochemical characterization techniques were employed to determine coordination modes, degradation process, and crystal structure. The luminescent and spectroscopic characteristics were evaluated through measurements of the emission and excitation spectra, lifetime, and quantum yield. Likewise, the CIE 1931 chromatic coordinates and color purity were determined to identify its colorimetric properties. Finally, the luminescent thermal stability of the complex was examined by analyzing the emission spectrum over the temperature range 30–150 °C.

## 2 Experimental Procedures

Europium­(III) nitrate pentahydrate (99.9%) (Eu­(NO_3_)_3_·5H_2_O), 1,10-Phenanthroline (99%) (Phen), and ethyl alcohol (99.9%) (EtOH) were obtained from commercial sources (Sigma-Aldrich) and were used as received without any additional steps.

### 2.1 Synthesis of the [Eu­(Phen)_2_(NO_3_)_3_] Complex

The [Eu­(Phen)_2_(NO_3_)_3_] complex was synthesized following a stoichiometric Phen:Eu^3+^ molar ratio of 2:1. Europium­(III) nitrate pentahydrate (50 mg, 0.11 mmol) was first dissolved in 5 mL of ethanol under continuous magnetic stirring at ambient temperature. In a separate vessel, 1,10-Phenanthroline (39.64 mg, 0.22 mmol) was dissolved in 5 mL of ethanol and then slowly added dropwise to the europium solution, leading to the immediate formation of a cloudy white suspension. The mixture was stirred for an additional 2 h and subsequently left to age in the dark for 24 h to promote complete precipitation. The resulting solid was collected by filtration using a Whatman No.1 filter paper (90 mm pore size) and washed three times with 20 mL of ethanol to remove residual unreacted species. Finally, the product was dried in a furnace at 85 °C for 24 h, obtaining a white solid with a 75% yield based on europium content, hereafter referred to as [Eu­(Phen)_2_(NO_3_)_3_].

### 2.2 Characterization Equipment

All the characterizations of the [Eu­(Phen)_2_(NO_3_)_3_] complex were performed in the solid state using a combination of structural, thermal, and optical techniques, including infrared spectroscopy, thermogravimetric analysis, powder X-ray diffraction, and photoluminescence measurements, together with temperature-dependent luminescence studies.

Infrared spectra were collected at room temperature over the 4000–400 cm^–1^ spectral range using a PerkinElmer Frontier FTIR/FIR spectrometer equipped with a universal attenuated total reflectance (ATR) accessory, operating at a resolution of 4 cm^–1^. The thermogravimetric curves were recorded over a temperature range of 50–800 °C under a nitrogen atmosphere flowing at 100 mL min^–1^, with a heating rate of 10 °C min^–1^, using a TA Instruments TGA Q5000 high-resolution thermobalance.

Crystallographic information was obtained from powder X-ray diffraction data recorded on a Bruker D8 Advance diffractometer employing CuKα radiation (λ = 0.1542 nm) in Bragg–Brentano configuration. The instrument operated without a primary-beam monochromator and used a Lynx Eye detector in the secondary beam. The diffraction pattern was measured over a 2θ interval of 5–70°, with a step size selected to ensure a minimum of eight points across the Full Width at Half Maximum (fwhm) of the principal reflections. Structural refinement was performed through the Le Bail method^21^ using the FullProf suite,^22^ allowing iterative optimization of lattice parameters, peak profiles, reflection positions, and intensities until convergence was achieved.

Photoluminescence measurements were performed using an Edinburgh Instruments FS5 spectrofluorometer equipped with a 150 W continuous-wave ozone-free xenon arc lamp and a Czerny-Turner monochromator incorporating a dual-grating turret. Emission spectra were obtained under fixed excitation at 350 and 396 nm, whereas excitation spectra were recorded by monitoring the characteristic Eu^3+^ emission at 615 nm. The instrumental parameters were adjusted with excitation and emission slit widths ranging from 0.20 to 1.00 nm, employing a spectral increment of 1.0 nm and an integration time of 0.10 s per point. Absolute photoluminescence quantum yield (Φ) was determined using an integrating sphere coupled to the same system. The luminescent decay curves were collected with a xenon flash lamp operating at a 3 μs per pulse, using a delay time of 0.01 ms and slits widths fixed at 1.5 nm. All steady-state optical measurements were conducted at room temperature.

Temperature-dependent luminescence experiments were also performed using the Edinburgh Instruments spectrofluorometer FS5, where the sample was mounted on a custom-built heating stage regulated by an IBEST TCM-series controller. The heating platform was connected through an optical fiber and carefully aligned with both excitation and emission inputs to maximize signal collection. For these measurements, the material was prepared in pellet form by compressing 50 mg of powdered sample within a cylindrical mold of 0.6 cm diameter, followed by the application of a 2-ton load for 2 min using a hydraulic press. The luminescence response was evaluated over a temperature interval from 30 to 150 °C, in steps of 10 °C.

## 3 Results and Discussion

The proposed red-emitting phosphor, [Eu­(Phen)_2_(NO_3_)_3_], was successfully synthesized using an efficient precipitation method, obtaining an instantaneous 75% yield. Several important characteristics of this compound were assessed, including the coordination environment, crystallinity, thermal stability, structural analysis, luminescence lifetime (τ), quantum yield (Φ), photometric analysis, and temperature-dependent luminescence properties. This article was conducted using FTIR, TGA, PXRD, and luminescence spectroscopy, as highlighted in the following sections.

### 3.1 FTIR Spectroscopy

With the objective of visualizing the formation of the europium complex, the FTIR spectra of Eu­(NO_3_)_3_·5H_2_O, 1,10-Phenanthroline, and the resulting europium complex are presented in Figure 1. Figure 1A displays the FTIR spectrum corresponding to Eu­(NO_3_)_3_·5H_2_O and reveals four distinct bands between 3600 and 3200 cm^–1^, which match the ν­(O–H) stretching bands. In addition, the δ­(O–H) bending at 1624 cm^–1^ with a shoulder at 1654 cm^–1^ confirms the existence of more than one water molecule.^23^ The other assigned bands are consistent with vibrational modes of the nitrate group, confirming their bidentate chelating coordination, which belongs to the distinctive vibrational bands for the nitrate ligands. The N=O symmetric stretching (ν_1_) was detectable at 1457 cm^–1^, accompanied by a shoulder near 1482 cm^–1^, while the out-of-plane deformation (ν_2_) was identified at 812 cm^–1^. The asymmetric NO_2_ stretching (ν_3_) was observed at 1290 cm^–1^, with additional shoulders at 1317 and 1273 cm^–1^, and the in-plane bending (ν_4_) at 746 cm^–1^, both of which are doubly degenerate. Lastly, the symmetric NO_2_ stretching mode (ν_5_) was detectable at 1025 cm^–1^, with a secondary shoulder at 1044 cm^–1^, further supporting the proposed coordination environment.^24–26^

**Figure 1:**
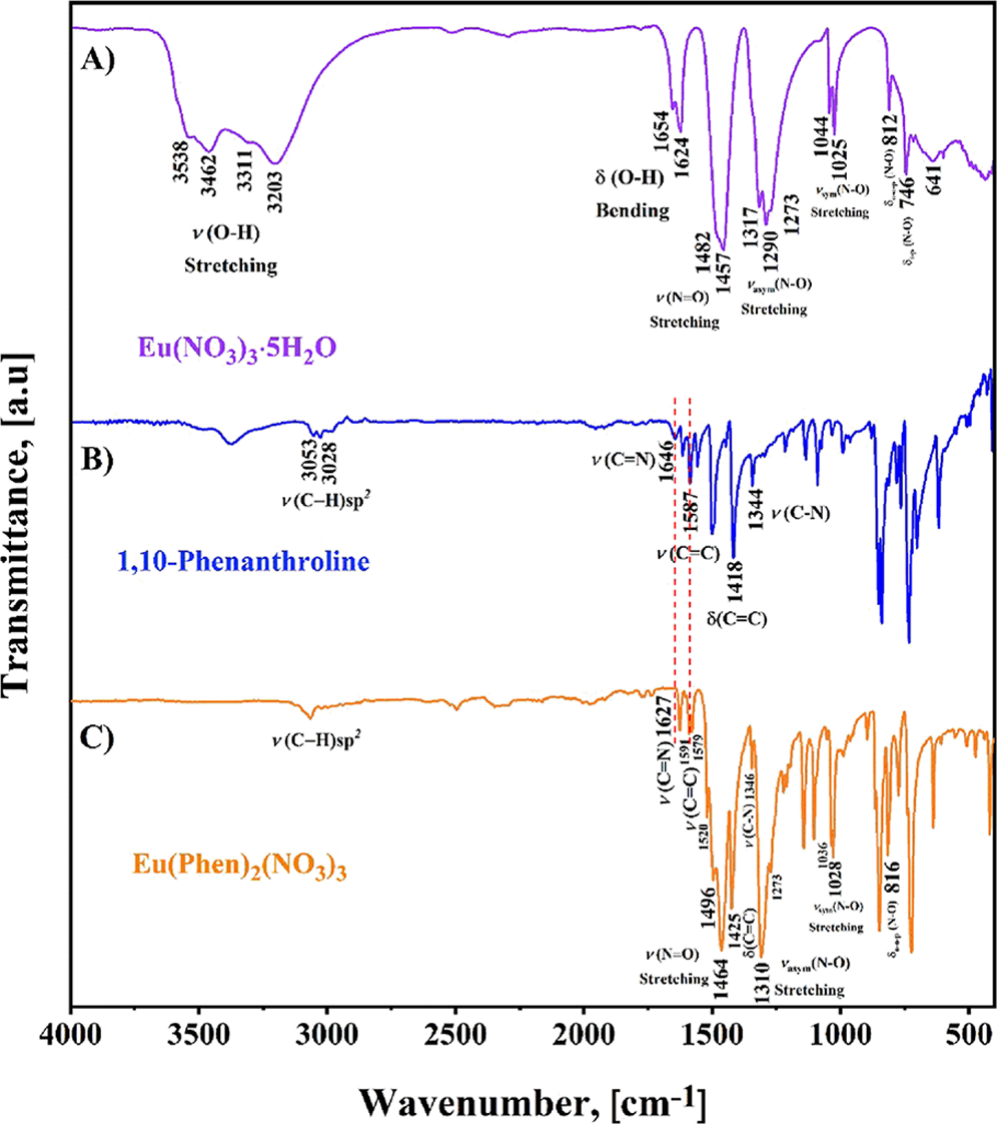
Comparative FTIR spectra recorded at room conditions for (A) the Eu­(NO_3_)_3_·5H_2_O, (B) the 1,10-Phenanthroline ligand, and C) the resulting [Eu­(Phen)_2_(NO_3_)_3_] complex.

Figure 1B illustrates the FTIR spectrum of the Phen ligand, in which two bands are observable at 1646 and 1587 cm^–1^ corresponding to aromatic stretching ν­(C=N) and ν­(C=C), respectively. Besides, the vibration modes assigned to the pyridine rings that structure the organic ligand were noticed at 1418 cm^–1^ for the δ­(C=C) and at 1344 cm^–1^ associated with ν­(C=N).

The principal vibrational frequencies of the [Eu­(Phen)_2_(NO_3_)_3_] complex are presented in Figure 1C, which differs significantly from the FTIR spectrum of the Phen ligand (See Figure 1B). First, the stretching frequency ν­(C=N) shifts from 1587 cm^–1^ in the free Phen to a double peak at 1591 and 1579 cm^–1^ upon complex formation. This fact suggests the possible presence of several modes of the Phen ligand in the coordination sphere, and a slight weakening of the C=N bond, which is the consequence of the electron donation from the nitrogen to the lanthanide ion. This effect causes a shift toward lower wavenumbers in the spectrum because the electron pairs from the donor nitrogen of the Phen ligand are attracted to the lanthanide ion, making them responsible for the coordination bonds (N → Eu^3+^). Furthermore, coordination-induced shifts were also detected for several vibrational modes associated with the Phen ligand, notably the ν­(C=C) stretching band moved from 1587 to 1579 cm^–1^, the δ­(C=C) deformation mode shifted from 1418 to 1425 cm^–1^, and the ν­(C–N) stretching vibration slightly changed from 1344 to 1346 cm^–1^. These shifts provide clear evidence of the undeniable coordination of the Phen ligand to the Eu^3+^ ion.

Another notable experimental data point in the FTIR spectrum of Figure 1C is the appearance of the N=O symmetric and N–O asymmetric stretching associated with the nitrate salt. The N=O symmetric stretching appears at 1464 cm^–1^, and the asymmetric NO_2_ stretching at 1310 cm^–1^. Both bands are shifted to higher wavelength numbers than the nitrate salt (1457 and 1290 cm^–1^), indicating coordination between the nitrate anion and the Eu^3+^ ion. In addition, the band at 816 cm^–1^ (Figure 1C) reveals that the nitrate is coordinated to Eu^3+^ through the bidentate site of the anionic structure.

Finally, it is relevant to mention that the resulting europium complex (Figure 1C) did not exhibit bands associated with O–H vibrations corresponding to EtOH or H_2_O. Therefore, it was possible to obtain an anhydrous phase of the Eu-complex. The absence of the O–H vibration is crucial for the performance of luminescence, as analyzed in the TGA and photoluminescence sections.

### 3.2 Thermogravimetric Analysis

Figure 2 displays the thermogram corresponding to the [Eu­(Phen)_2_(NO_3_)_3_] complex within the temperature range of 50–800 °C, illustrating both the TGA curve (represented by the red line) and the DTG curve (depicted as an orange dotted line). In correspondence with the evidence obtained from the FTIR spectrum (See Figure 1C), no weight loss is appreciated upon heating the sample below 125 °C. This fact is desirable in lanthanide complexes, as the anhydrous phase may be more efficient in its luminescent performance, since OH vibrations are known to quench luminescence.^27^ The first decomposition begins at ∼390 °C with the degradation of the organic ligands and nitrate anions, reaching its peak decomposition rate at 413 °C and resulting in a weight loss of 72%. This percentage is associated with the initial decomposition process involving two Phen ligands and two nitrate anions. It is important to highlight the evolution of the organic ligand around 100 °C above the degradation temperature as a free 1,10-Phenanthroline at 250–350 °C (see Figure S1), which confirms its coordination with the Eu^3+^ ion. The last degradation process occurred gradually within the 650–800 °C range and possibly after, contributing to a 6% weight loss corresponding to the final nitrate anion. These assignments correspond to a usual decacoordination of europium, suggesting that the two Phen ligands and three nitrate anions may be coordinated to the europium ion. Here, the Phen and the nitrates should be in a bidentate coordination mode, further confirmed by the structural analysis presented in the following section. Lastly, the remaining 22% of the weight is attributed to a single europium ion, thereby finalizing the composition of the [Eu­(Phen)_2_(NO_3_)_3_] compound. Therefore, the data obtained from the TGA were essential for determining the experimental formula unit of the material under investigation, identified as [Eu­(Phen)_2_(NO_3_)_3_].

**Figure 2:**
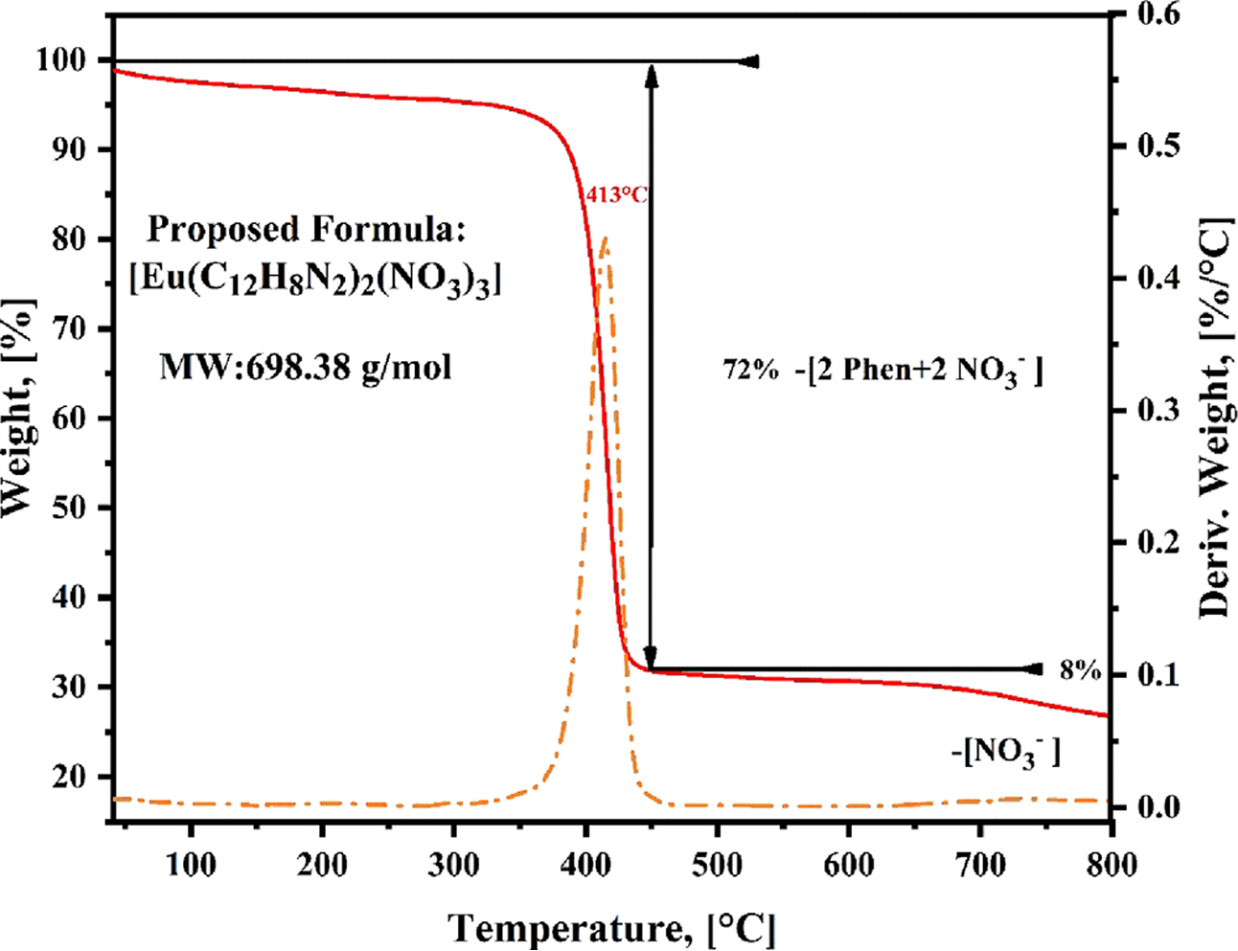
TGA curve and DTG curve of the resulting europium complex with the proposed formula unit [Eu­(Phen)_2_(NO_3_)_3_].

### 3.3 PXRD Study and Le Bail Fitting

The crystal structure of the synthesized [Eu­(Phen)_2_(NO_3_)_3_] complex was examined through powder X-ray diffraction analysis. To interpret the data, a Le Bail refinement was performed by matching the experimental powder diffractogram with a simulated pattern derived from the crystallographic model previously reported by Sadikov et al., archived in the Cambridge Crystallographic Data Centre (CCDC, deposition number 252725).^17^ Figure 3A presents the profile fitting performed using the FULLPROF software,^22^ where experimental intensities data are shown as red markers, the calculated pattern appears as a solid black curve, the residual difference is plotted in blue, and the Bragg reflection positions associated with the reference structure are indicated in green thicks. The strong agreement between the measured and simulated patterns confirms that the synthesized material preserves the same structural symmetry and unit cell metrics as the reported phase. The complex crystallizes in a monoclinic lattice with space group *C*2/*c* (15), containing four formula units per cell (*Z* = 4), and exhibits cell parameters of *a* = 9.5153 Å, *b* = 15.4546 Å, *c* = 17.1763 Å, β = 93.451°, and a unit cell volume of 2521.28 Å^3^. Furthermore, the coordination environment of the europium ion is illustrated in Figure 3B, resulting in a decacoordinated sphere consisting of four nitrogen atoms from two 1,10-Phenanthroline molecules (4-N_Phen_) coordinated in a bidentate chelating mode and six oxygen atoms from three nitrate anions (6-O_NO_3__) coordinated in an isobidentate mode. These results confirm the preliminary information obtained from the FTIR and TGA.

**Figure 3:**
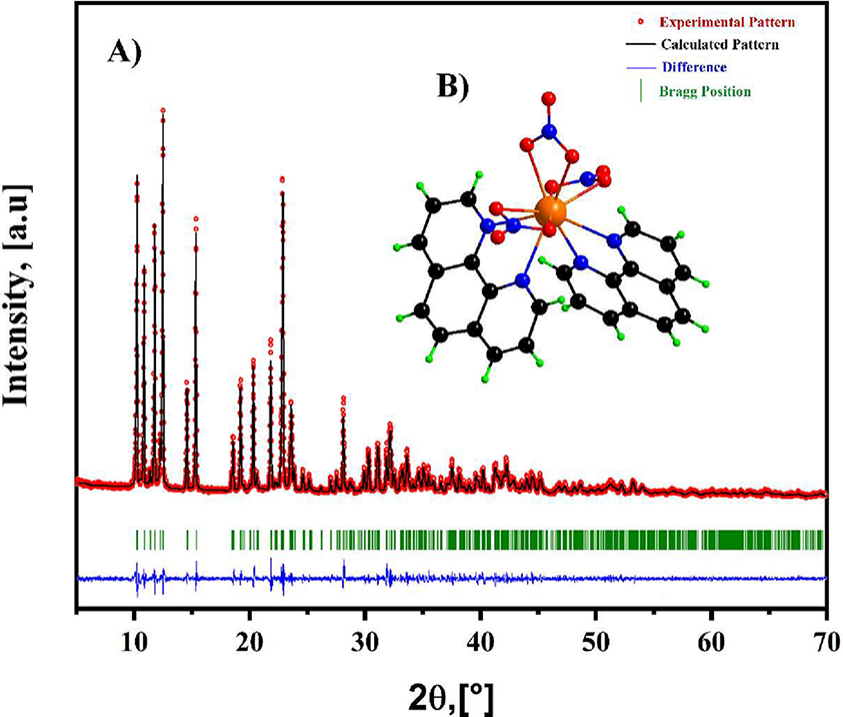
(A) The PXRD diffractogram of the [Eu­(Phen)_2_(NO_3_)_3_] complex refined by the Le Bail method; (B) the asymmetric unit displays the decacoordinated environment of the Europium ion.

### 3.4 PL Properties of the [Eu­(Phen)_2_(NO_3_)_3_] Complex

Figure 4 presents the excitation and emission photoluminescence spectra of the [Eu­(Phen)_2_(NO_3_)_3_] system. The excitation spectrum was measured by tracking the strongest Eu^3+^ radiative transition, corresponding to ^5^D_0_ → ^7^F_2_ emission at 615 nm (Figure 4A). The resulting spectrum displays two prominent broad absorption regions centered at approximately 256 and 350 nm, which are attributed to ligand-to-metal charge transfer (LMCT) processes and the π → π* electronic transitions of the Phen ligand, respectively.

**Figure 4:**
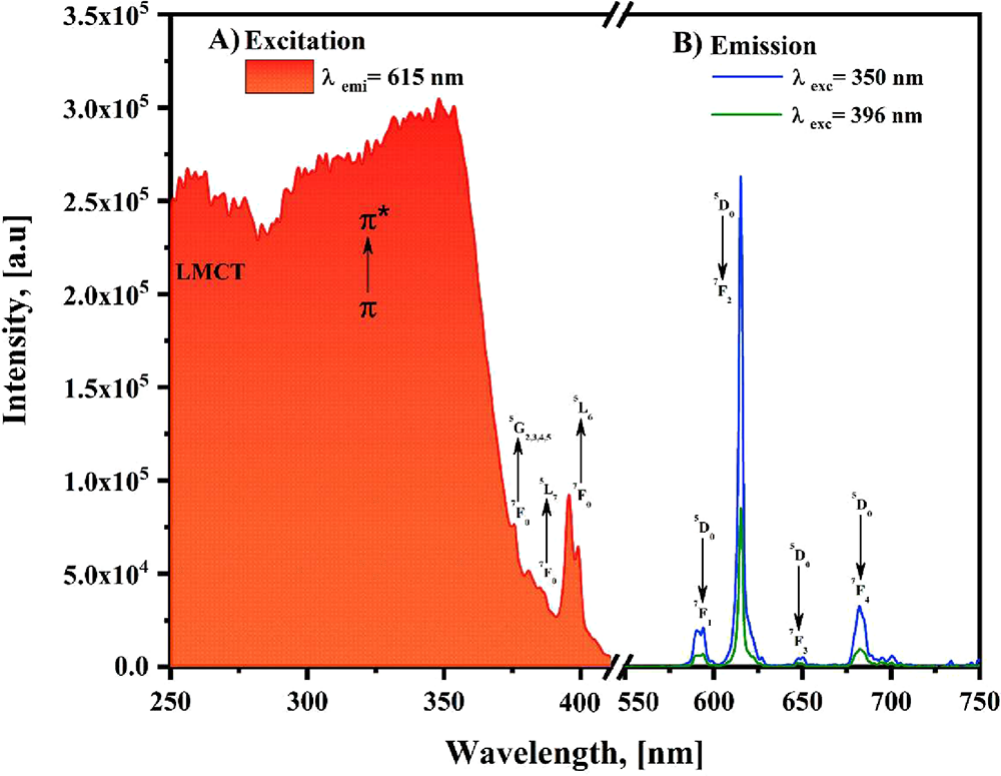
(A) The excitation spectrum of the [Eu­(Phen)_2_(NO_3_)_3_] complex monitoring at λ_emi_ = 615 nm; and (B) the emission spectrum obtained at λ_exc_ = 350 and 396 nm, both at room temperature.

A high-energy band centered at ∼256 nm is attributed to ligand-to-metal charge transfer (LMCT) transitions involving the Phen ligand and the Eu^3+^ ion. Such LMCT states are commonly observed in Eu^3+^ coordination compounds and could contribute to the population of excited states at higher excitation energies.^13,28^ Furthermore, the broader and more intense excitation band around 350 nm is mainly assigned to the π → π* transition of the phenanthroline ligand. This band efficiently sensitizes the Eu^3+^ emission through ligand-to-metal energy transfer, known as the antenna effect. Owing to its higher intensity and favorable excitation window (320–370 nm), this pathway represents the predominant photophysical mechanism responsible for the observed Eu^3+^ luminescence in the [Eu­(Phen)_2_(NO_3_)_3_] complex.^29^

Additionally, several parity-forbidden intra-*4f* transitions corresponding to the Eu^3+^ ion are exhibited as follows: ^7^F_0_ → ^5^G_2,3,4,5_ (376 nm), ^7^F_0_ → ^5^L_7_ (381 nm), and ^7^F_0_ → ^5^L_6_ (396 nm).^11^ The observation of bands associated with the Phen ligand π → π* transition, together with the characteristic parity-forbidden *4f–4f* transitions, suggests that Eu^3+^ luminescence may arise through two distinct excitation pathways^13^: (1) Sensitization via energy transfer from the coordinated organic ligand to the lanthanide center, and (2) Direct excitation of the Eu^3+^ ion itself. Notably, the intense broad bands corresponding to the Phen ligand indicate that the excitation of the [Eu­(Phen)_2_(NO_3_)_3_] complex is more effectively achieved through the antenna effect of the organic ligand than pathway the direct excitation mechanism of the Eu^3+^.

Figure 4B displays the emission spectra obtained after excitation at λ_exc_ = 350 nm and λ_exc_ = 396 nm. First, with a λ_exc_ = 350 nm, multiple emission bands related to the intraconfigurational *4f* ^6^ *–4f* ^6^ transitions characteristic of the Eu^3+^ ion were noticed at 590 nm (^5^D_0_ → ^7^F_1_), 615 nm (^5^D_0_ → ^7^F_2_), 650 nm (^5^D_0_ → ^7^F_3_), and 682 nm (^5^D_0_ → ^7^F_4_).^11^ The absence of emissions associated with the Phen ligand highlights the energy transfer efficiency from the Phen ligand to the Eu^3+^ ions.^30^ The magnetic dipole transition (^5^D_0_ → ^7^F_1_) and the induced electric dipole transition (^5^D_0_ → ^7^F_2_) provide additional insights into the coordination environment in Eu^3+^ compounds.^31,32^ For instance, the presence of a splitting in the ^5^D_0_ → ^7^F_1_ transition band may suggest a magnetic dipole interaction due to the ligand effects. Although the ligand field does not strongly influence the magnetic dipole transition compared to electric dipole transitions, the splitting observed directly results from the ligand field’s strength and asymmetry acting on the ^7^F_1_ state. In the emission spectrum (see Figure 4B), the most intense band corresponds to the hypersensitive electric dipole transition from ^5^D_0_ → ^7^F_2_, which is responsible for the characteristically red pure emission in materials containing Eu^3+^ ions.^33^ Additionally, this transition is characterized by the narrowest line width and the lowest intensity ever reported, rendering it undetectable, as observed in this instance.^11^

In accordance with the Judd-Ofelt theory, the integrated intensity ratio of the electric and magnetic dipole transitions (^5^D_0_ → ^7^F_2_/ ^5^D_0_ → ^7^F_1_) offers an insight into the degree of distortion from the inversion symmetry of the system.^34^ The intensity ratio ^5^D_0_ → ^7^F_2_/ ^5^D_0_ → ^7^F_1_ for the [Eu­(Phen)_2_(NO_3_)_3_] complex, measured under excitation at 350 nm, was determined to be 6.4. Values greater than 1 suggest a high distortion grade in the local site symmetry around the Eu^3+^ ion, consistent with the structural analysis results (see Figure 3B).

On the other hand, with a direct excitation of λ_exc_ = 396 nm, the [Eu­(Phen)_2_(NO_3_)_3_] complex exhibited the characteristic ^5^D_0_ → ^7^F_*J*_ (*J* = 1–4) transitions, consistent with the emissions observed at λ_exc_ = 350 nm. Remarkably, the appearance of the emission from the ^5^D_1_ states suggests efficient nonradiative relaxation from the triplet level of the Phen ligand to the ^5^D_1_ and ^5^D_0_ states, followed by radiative transitions to the ^7^F_j_ levels (See Figure S2).

Based on the experimental results of PL, Figure 5 presents the energy diagram of the [Eu­(Phen)_2_(NO_3_)_3_] complex with λ_exc_ = 350 nm and λ_exc_ = 396 nm. The energy transfer mechanism from the ligand to the metal center can be described as follows: When the complex is irradiated at 350 nm (28,571 cm^–1^), electrons are promoted from the singlet ground state (S_0_) to a higher-energy excited singlet state (S_1_). Subsequently, intersystem crossing (ISC) occurs, facilitating the transition of electrons from the S_1_ state to the triplet state (T_1_) at 22,100 cm^–1^.^4^ It is noteworthy to mention that the proposed energy transfer mechanism should comply with empirical criteria proposed by Reinhoudt and Latva, which indicate that ligand-to-lanthanide energy transfer is efficient when the gap between the ligand triplet state and the receiving excited level of the lanthanide ion lies approximately 2000–4000 cm^–1^.^35,36^ Considering those mentioned above, since the Eu^3+^ excited states ^5^D_1_ (18,973 cm^–1^) and ^5^D_0_ (17,227 cm^–1^) are positioned at lower energies than the triplet state of the Phen, the energy transfer could occur by two viable mechanisms: (1) Starting from the T_1_ level to the ^5^D_1_ level, where internal conversion proceeds to ^5^D_0_. Then, from the ^5^D_0_ level, emissions to the ^7^F_*J*_ manifolds are exhibited. (2) Directly going from the T_1_ to the ^5^D_0_ level, followed by the emission to the ^7^F_J_ manifolds. It is considered that the first mechanism, T_1_ → ^5^D_1_, is more favorable due to the energy gap between the two levels being 3127 cm^–1^, which falls within the range of the empirical rule.

**Figure 5:**
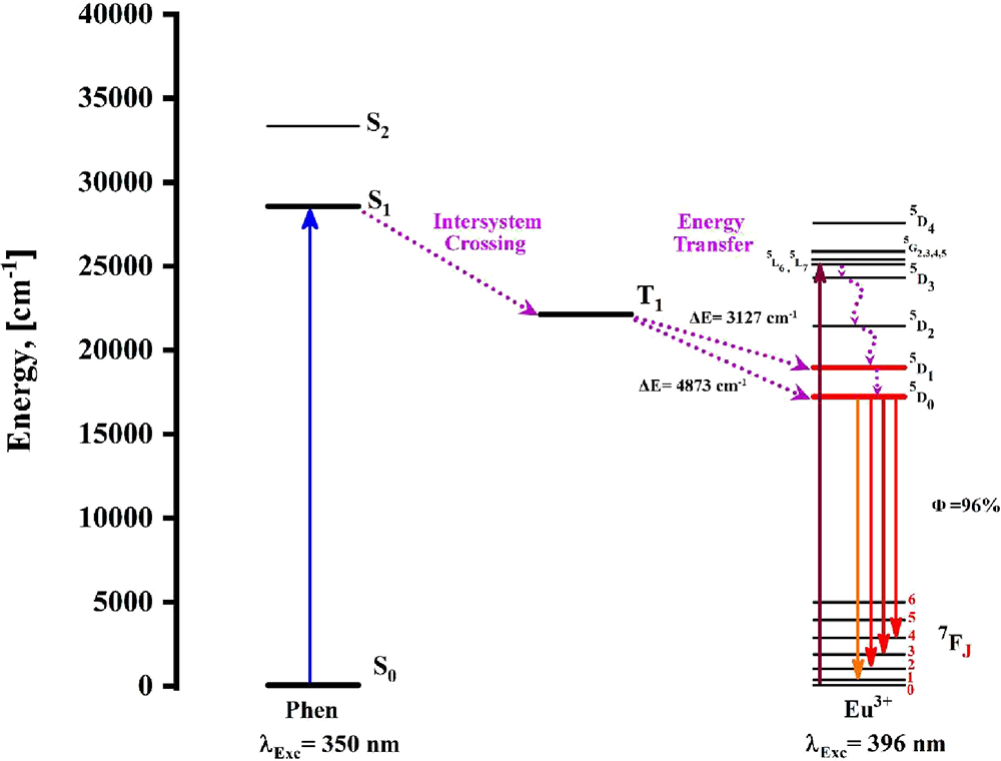
Energy level diagrams of the Phen ligand and Eu^3+^, including the proposed energy transfer mechanism.

Focusing on the direct excitation of the Eu^3+^ ion at 396 nm (^7^F_0_ → ^5^L_6_), which is responsible for the emissions from the ^5^D_0_ → ^7^F_1–4_ transitions, could arise through the following mechanism: When excited at 396 nm, electrons are promoted from the ^7^F_0_ ground state to the ^5^L_6_ excited level. Subsequently, they undergo nonradiative relaxation from ^5^L_6_ to the ^5^D_0_ level, from which they radiatively decay to the ^5^D_0_ → ^7^F_1–4_ manifolds. This interpretation is supported experimentally by the absence of emission transitions associated with the ^5^D_3_ and ^5^D_2_ energy levels, which are detected within the 500–560 nm range.

Another relevant feature of the emission spectrum is the experimental branching ratios (β_R_) that describe the spectral distribution of emission intensity for each transition.^37^ If we consider the Eu^3+^ emission presented in Figure 4, the β_R_ of [Eu­(Phen)_2_(NO_3_)_3_] complex could be computed as follows:

$$\beta_{R}{(}^{5}D_{0}\to^{7}F_{J})=\frac{A{(}^{5}D_{0}\to^{7}F_{J})}{A_{R}{(}^{5}D_{0})}$$ 1

Where *A* is the radiative transition probability (*A*), and *A*_R_ is the total radiative transition probability. The experimental β_R_ for the ^5^D_0_ → ^7^F_1–4_ transitions are 10, 63, 3, and 21%, respectively. With a negligible contribution of less than 3%, they correspond to level ^5^D_1_. As can be seen, the ^5^D_0_ → ^7^F_2_ transition exhibits a β_R_ above 60% and an fwhm of 3.1 nm, making this feature ideal as an active optical medium in laser devices^38^ and the generation of LEDs and OLEDs for lighting systems.^39,40^

### 3.5 Lifetime and Quantum Yield

The photoluminescence lifetime (τ) decay of the [Eu­(Phen)_2_(NO_3_)_3_] complex was evaluated under 350 nm while tracking the hypersensitive Eu^3+^ emission centered at 615 nm. The resulting decay profile is presented in Figure 6, and is exponentially fitted according to the reported equation^41^:

$$I(t)=I_{0}Ae^{-t/\tau }$$ 2

In this context, *I*_0_ represents the initial intensity, *A* is a constant, *t* is the time, and τ is the lifetime. When the complex is under excitation at 350 nm and monitored at λ_emi_ = 615 nm, the decay curve exhibits a monoexponential behavior with a decay time (τ) of 1.096 ± 0.001 ms, with χ^2^ = 1.006 and a coefficient of determination of *R* ^2^ indicating good fitting. The residuals are randomly distributed around zero with no systematic deviations, confirming the adequacy of the model (See Figure S3). This monoexponential decay behavior indicates a single coordination environment around the Eu^3+^ ion in the [Eu­(Phen)_2_(NO_3_)_3_] complex, consistent with four formula units (*Z* = 4), as observed in the monoclinic cell obtained by Le Bail refinement (See Figure S4). The lanthanide complexes exhibited more efficient radiative pathways and a decrease in nonradiative transitions compared to europium-free compounds in their salt form, as documented for Eu­(NO_3_)_3_·5H_2_O in the literature, which has a lifetime of around 0.1–0.5 ms, depending on the environment.^42^

**Figure 6:**
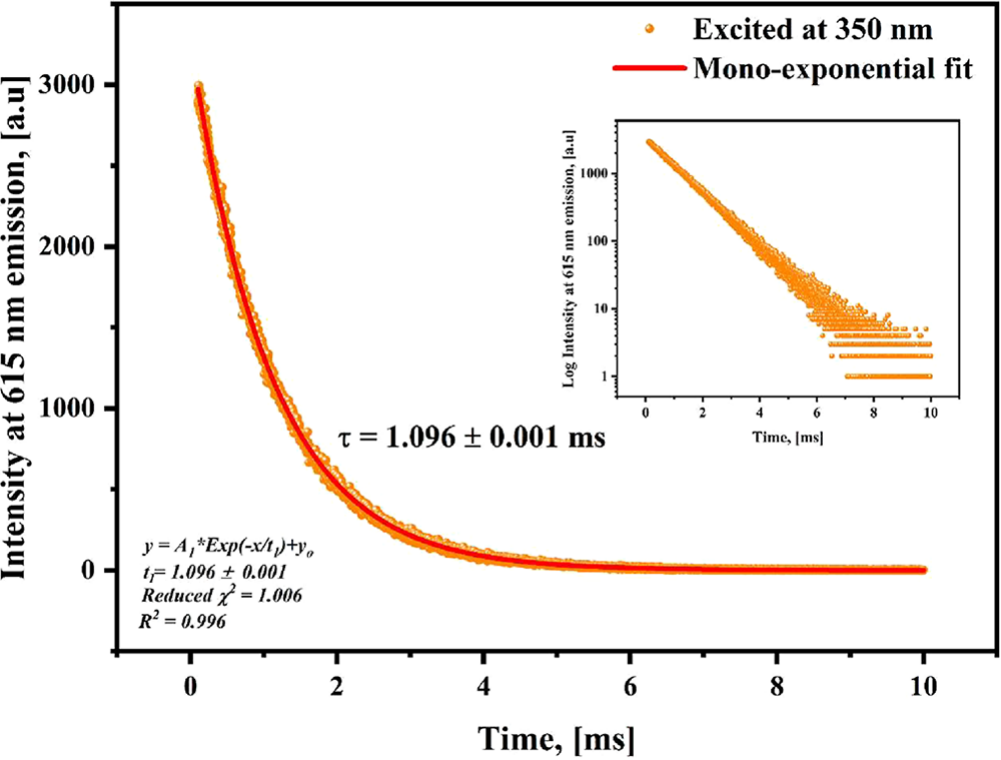
Luminescence decay profile of [Eu­(Phen)_2_(NO_3_)_3_] complex recorded with λ_exc_ = 350 nm and monitored at λ_emi_ = 615 nm.

The luminescence lifetime obtained in this work (τ = 1.096 ± 0.001 ms) is comparable to or higher than those reported for several Eu^3+^ coordination compounds in the recent literature. For instance, Malik et al. reported that brominated β-diketonate europium­(III) complexes exhibit lifetimes ranging from 0.56 to 0.90 ms, depending on ligand substitution and coordination symmetry.^43^ Similarly, macrocyclic Eu^3+^ complexes reported by Salazar-Medina et al. exhibit lifetimes of approximately 0.65 ms.^44^ In the case of Eu^3+^ complexes based on phthalic acid ligands reported by Guzman-Silva et al., lifetimes ranging from 0.49 to 1.67 ms are strongly influenced by the presence of coordinated water molecules.^45^ Additionally, Li et al. presented Eu^3+^ complexes with ligands, such as ((1*E*,1′*E*)-*N*,*N*′-(1,2-diphenylethane-1,2-diyl)­bis­(1-(pyridin-2-yl)­methanimine)), which display luminescence lifetimes of approximately 1109.7 μs (1.109 ms),^46^ which is essentially identical to the value obtained for the present system, but with lower quantum yields. These facts demonstrate the superior performance of the [Eu­(Phen)_2_(NO_3_)_3_] complex via radiative pathway transitions, suggesting that the coordination of the Eu^3+^ ion to the Phen ligand enhances radiative efficiency, which is attributed to the antenna effect.^47^

To evaluate the enhancement of the radiative pathways in the complex, the absolute quantum yield (Φ) was determined utilizing an integrating sphere, applying the following formula^48^:

$$\Phi =\frac{\int F_{c}}{\int L_{a}-\int L_{c}}$$ 3

In this analysis, *F*_c_ represents the emission spectrum of the complex, *L*_a_ corresponds to the excitation spectrum in the absence of the sample, and *L*_c_ denotes the excitation signal in the presence of the europium complex. The difference *L*_a_ *–L*_c_ quantifies the total photon flux absorbed by the material. Using an excitation wavelength of 350 nm, the [Eu­(Phen)_2_(NO_3_)_3_] complex exhibited an absolute photoluminescence quantum yield (Φ) of 96%. A high quantum yield is ideal for applications such as red-emitting LEDs, bioimaging, Lasers, and optical sensors, as it ensures uniform, intense luminescence.^49^ This is particularly valuable for thermal sensors and lighting devices operating in demanding conditions that require stable, critical emission performance.^50^

It is crucial to emphasize that the ligand–metal energy-transfer process exhibits a high quantum yield.^35^ The absolute quantum yield (Φ) value obtained for our europium complex surpasses those of different (Irpiq) complexes (Φ = 17–32%)^51^ and certain inorganic compounds, including the commercially available phosphor Y_2_O_3_:Eu^3+^ (Φ = 9.6%)^52^ and red-emitting CdSe/CdS/ZnS QDs (Φ > 90%).^53^

### 3.6 Photometric Analysis

Given the promising luminescent properties of the [Eu­(Phen)_2_(NO_3_)_3_] complex, the CIE 1931 chromatic coordinates and color purity were examined to evaluate its performance and its potential as a red-emitting phosphor. The emission spectra obtained at λ_exc_ = 350 nm, as shown in Figure 4B, was utilized for this photometric analysis.

Figure 7 illustrates the CIE 1931 chromaticity diagram for the overall emission of the [Eu­(Phen)_2_(NO_3_)_3_] complex, which exhibits chromaticity coordinates of (0.66, 0.33) when excited at 350 nm, corresponding to a deep-red luminescence. These values approximate the reference chromaticity coordinates established for an ideal red phosphor by the European Broadcasting Union (EBU) standard (0.64, 0.33),^54^ the National Television Standard Committee (NTSC) illuminant red phosphor (0.67, 0.33),^55^ red-emitting CdZnSe/ZnSe QDs (0.63, 0.37),^56^ and iridium complexes proposed as OLEDs (0.66, 0.33).^51^

**Figure 7:**
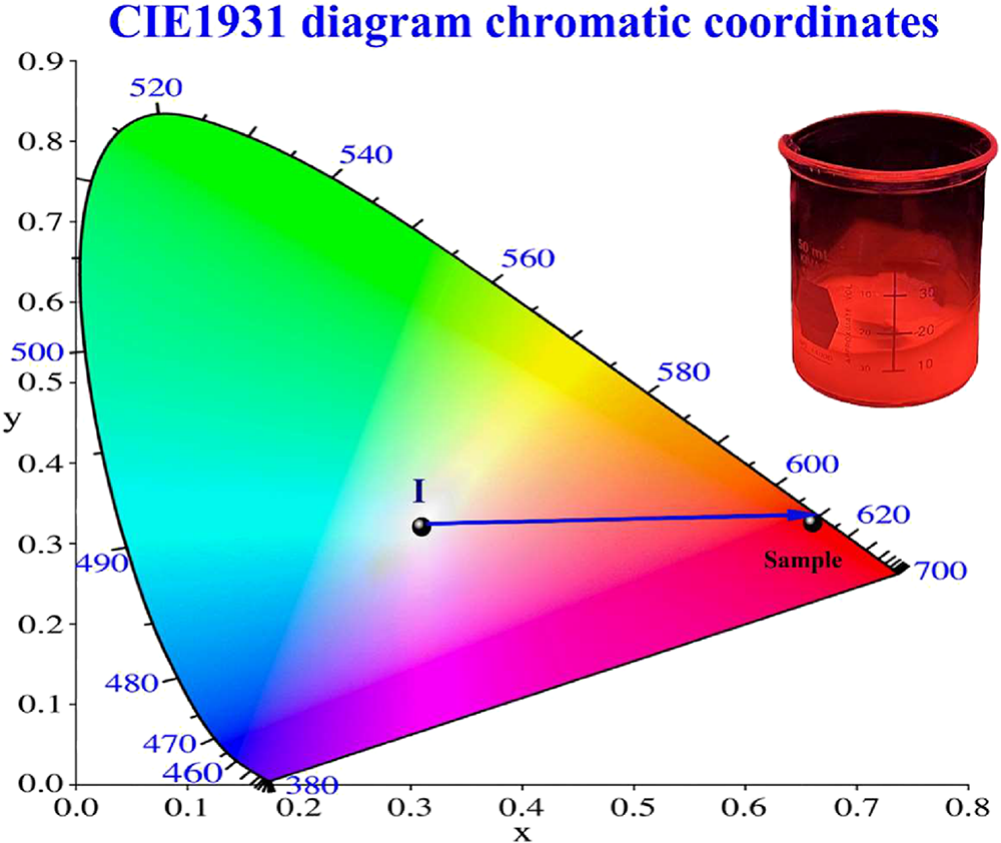
CIE 1931 chromatic coordinates of the [Eu­(Phen)_2_(NO_3_)_3_] complex, λ_exc_ = 350 nm, with an inset photograph of the red emission of the europium complex.

The calculation of color purity (CP) was performed using the following equation^57^:

$$\mathrm{CP}=\sqrt{\frac{(x-x_{i}{)}^{2}+(y-y_{i}{)}^{2}}{(x_{d}-x_{i}{)}^{2}+(y_{d}-y_{i}{)}^{2}}}$$ 4

where *x* and *y* represent the chromatic coordinates, while *x*_1_ and *y*_1_ correspond to the coordinates of the standard source *C* (0.31, 0.31),^58^ which is denoted as the position I in Figure 7. The values *x*_d_ and *y*_d_ refer to the dominant chromatic coordinates. Upon excitation at a wavelength of 350 nm, the sample exhibited a high color purity of 99%, with chromaticity coordinates of *x* = 0.66, *y* = 0.33. The corresponding dominant wavelength (λ_d_) was approximately 610 nm, as illustrated in Figure 7.

The inset in Figure 7 presents a photographic image of [Eu­(Phen)_2_(NO_3_)_3_] complex exhibiting red luminescence under irradiation with a 360 nm UV lamp. Meanwhile, Table 1 provides the CIE 1931 chromaticity coordinates determined for the [Eu­(Phen)_2_(NO_3_)_3_] complex, along with a comparative analysis against recent research findings on inorganic and organic materials exhibiting red emission, including color purity, quantum yield, their thermal stability at 150 °C, and their activation energy.

**Table 1:** Reported Red-Emitting Phosphor Compounds/Materials and Their Comparison with the Investigated [Eu­(Phen)_2_(NO_3_)_3_] Complex in This Work

phosphor	chromatic coordinates	CP (%)	Φ (%)	Δ*E* (eV)	thermal stability at 150 °C (%)	reference
Na_2_Gd(PO_4_)(MoO_4_):Eu^3+^	0.65, 0.34	92	37	0.22	63.3	^59^
GdBO_3_:Eu^3+^	0.60, 0.34		78	0.26	98	^60^
Ca_0.94_GeO_3_: 6%Eu^3+^	0.63, 0.33	99	30.5	0.29	82	^61^
Rb_2_Bi(PO_4_)(WO_4_):Eu^3+^	0.64, 0.35	91.1	88.1	0.23	98.6	^62^
[Eu_2_DPA_3_]_n_ MOF	0.65, 0.35	98.5	55	0.21	96.5	^63^
Sr_2_GdTaO_6_: Eu^3+^	0.61,0.36	3	94	0.24	57.6	^64^
Y_2_Mo_4_O_15_:Eu^3+^	0.64, 0.34	98		0.21	43.3	^65^
CsBSi_2_O_6_: Eu^3+^	0.56,0.42	75.9	9.42	0.18	80	^66^
Sr_2_Mg_3_P_4_O_15_:Eu^3+^	0.57,0.37			1.42	61	^67^
Ca_19_M_2_(PO_4_)_15_:Eu^3+^	0.65,0.34	99		0.19	73	^68^
[Eu(Phen)_2_(NO_3_)_3_]	0.66, 0.33	99	96	ZTQ	100	this work

It is noteworthy to emphasize that red emission with CIE values near the standards is essential for achieving high color purity, optimizing color rendering, enhancing visual comfort, and minimizing color distortion, resulting in a more visually appealing and comfortable lighting experience.^69,70^ Furthermore, optimizing the red emission component in WLEDs is crucial for achieving high luminous efficacy and minimizing power consumption, contributing to energy efficiency and reducing the environmental impact of artificial lighting.^71^

Notably, the performance and the organic nature of the [Eu­(Phen)_2_(NO_3_)_3_] complex exhibit promising characteristics for its potential use in organic light-emitting diodes (OLEDs).^40,72^ For instance, red-emitting lanthanide complexes can be effectively excited by near-ultraviolet (UV) or blue LEDs (400–470 nm), making them ideal for high-quality lighting systems, display technology, plant growth lighting, and bioimaging applications.

### 3.7 Zero Thermal Quenching

Since temperature significantly influences luminescence efficiency, evaluating the luminescence performance as a function of temperature is essential. Luminescent thermal stability is a crucial characteristic of phosphors intended for LEDs, OLEDs, or various lighting system applications. For this reason, a temperature-dependent analysis of the luminescence response was conducted on the [Eu­(Phen)_2_(NO_3_)_3_] complex, where the temperature varied between 30 and 150 °C.

Figure 8 presents the emission spectra of the europium complex excited at 350 nm. The spectra exhibited no substantial variations in the emission signals, except for a minor reduction in the intensity of the ^5^D_0_ → ^7^F_2_ transition as the temperature increased. Also, all the bands preserve the same shape with no wavelength shifts.

**Figure 8:**
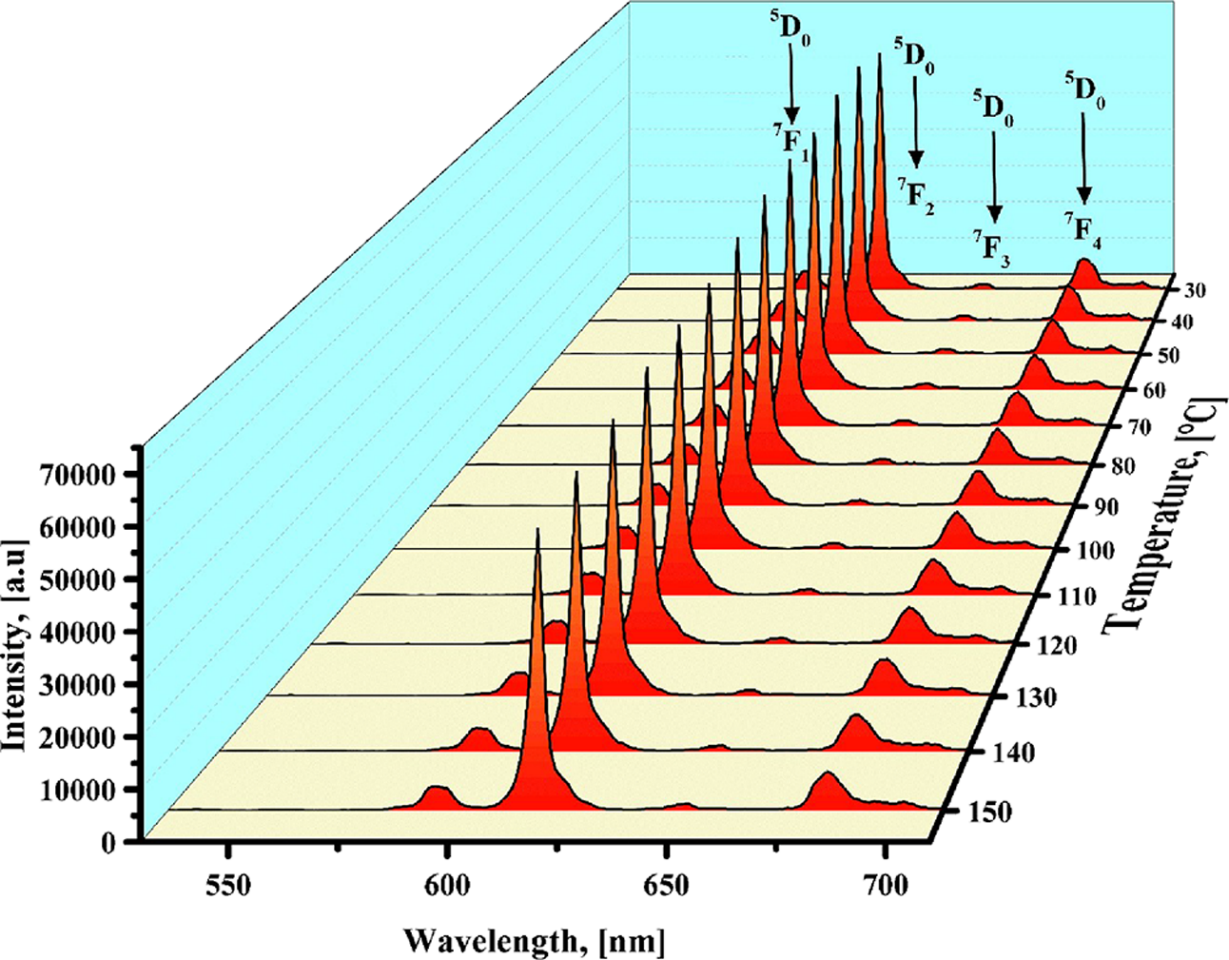
Temperature-dependent emission spectra of [Eu­(Phen)_2_(NO_3_)_3_] complex recorded between 30 and 150 °C under λ_exc_ = 350 nm.

To evaluate the temperature’s influence on the emission intensity of the [Eu­(Phen)_2_(NO_3_)_3_] complex, the normalized integrated photoluminescence intensity across the entire spectral emission range was calculated as a function of temperature, ranging from 303 to 423 K (30 to 150 °C). Figure 9 illustrated the integrated intensity values exhibited a notable increase of 1–6% at elevated temperatures. In particular, the overall spectral signal at 150 °C was 1% higher than its value at 30 °C, indicating an absolute thermally stable behavior characteristic of Zero Thermal Quenching (ZTQ).^73^

**Figure 9:**
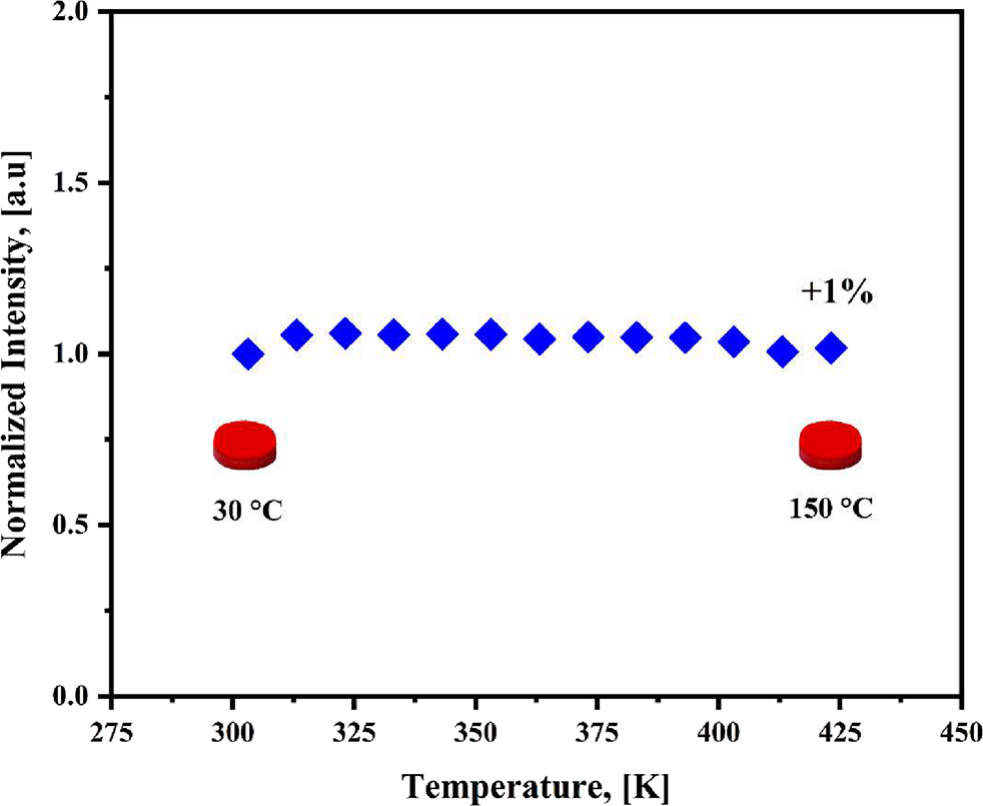
Variation of the normalized integrated emission intensity of the [Eu­(Phen)_2_(NO_3_)_3_] complex as a function of temperature (30–150 °C) under 350 nm excitation.

Thermal quenching is a widely observed phenomenon in which temperature increases. This effect limits the efficiency of materials in optoelectronic applications.^74^ It is desirable that the materials present ZTQ, where the emission intensity remains nearly constant or even increases with rising temperature.^75^ This result demonstrates the [Eu­(Phen)_2_(NO_3_)_3_] complex’s ability to be proposed as a thermally stable phosphor. This stability could be attributed to a large energy gap between the Phen ligand’s triplet state and the ^5^D_0_ state of the Eu^3+^ (4873 cm^–1^), which inhibits thermal back energy transfer (BET) from ^5^D_0_ to the T_1_ state.

The occurrence of BET in europium complexes has been both theoretically predicted and experimentally confirmed; it takes place when the excited europium ion transfers energy back to the ligand, leading to nonradiative deactivation instead of luminescence.^76,77^ This process is governed by the energy gap between the triplet state of the Phen ligand (T_1_) and the europium ^5^D_0_ excited level. When T_1_ is placed too close to ^5^D_0_ (less than 2000 cm^–1^), thermal energy could facilitate reverse energy transfer, depopulating ^5^D_0_ and reducing emission intensity, a phenomenon known as thermal quenching. On the other hand, if T_1_ is located too far from ^5^D_0_ (greater than 5000 cm^–1^), energy transfer from the ligand to Eu^3+^ is inefficient, resulting in weak luminescence. An optimal energy gap, typically ranging between 2000 and 4000 cm^–1^ as the empirical rule mentioned above, ensures efficient energy transfer to Eu^3+^ while preventing significant BET.^14,30,42^ Kumari et al. reported a series of six europium complexes, in which this phenomenon was analyzed and experimentally demonstrated. The complexes incorporating 2,2′-bipyridyl and Neocuproine ligands, characterized by Δ*E* (T_1_ – ^5^D_0_) of 5736 and 5465 cm^–1^, respectively, exhibited lower luminescence intensity compared to those coordinated with Phen. In contrast, the complexes containing Bathophenanthroline and 5,6-dimethyl-1,10-phenanthroline ligands also presented reduced luminescence intensity, likely due to the small energy gap between the T_1_ and the ^5^D_0_ level (3836 and 3933 cm^–1^), which may have promoted the BET process.^78^

In this work, the adequate energy gap of 4783 cm^–1^ between the triplet state of the Phen ligand and the ^5^D_0_ state of the Eu^3+^ could be a crucial factor contributing to zero thermal quenching by suppressing back energy transfer. At room temperature, the available thermal energy (E ≈ *k*_B_ *T*) is approximately 200 cm^–1^; even at high temperatures (150 °C), it reaches only around 300 cm^–1^. This energy is significantly lower than the 4783 cm^–1^ gap, making thermal repopulation of the Phen ligand’s triplet state from ^5^D_0_ present a low probability. Consequently, luminescence remains strong even at high temperatures, resulting in zero thermal quenching.

Another experimental observation was the enhancement of the ^5^D_1_ emission with increasing temperature. As shown in Figure 8, a closer examination of the 520–570 nm range reveals that the ^5^D_1_ → ^7^F_1_ and ^5^D_1_ → ^7^F_2_ transitions increase with rising temperature (see Figure 10A). The energy difference between ^5^D_0_ and ^5^D_1_ of the Eu^3+^ ion is 1746 cm^–1^; they could be considered as thermally coupled levels (TCL),^79^ and the ^5^D_1_ state can be populated as the temperature increases. Boltzmann’s formula determines the population of the two TCLs^80^:

$$\frac{I_{1}}{I_{2}}=B\exp\left(\frac{\Delta E}{k_{b}T}\right)+C$$ 5

**Figure 10:**
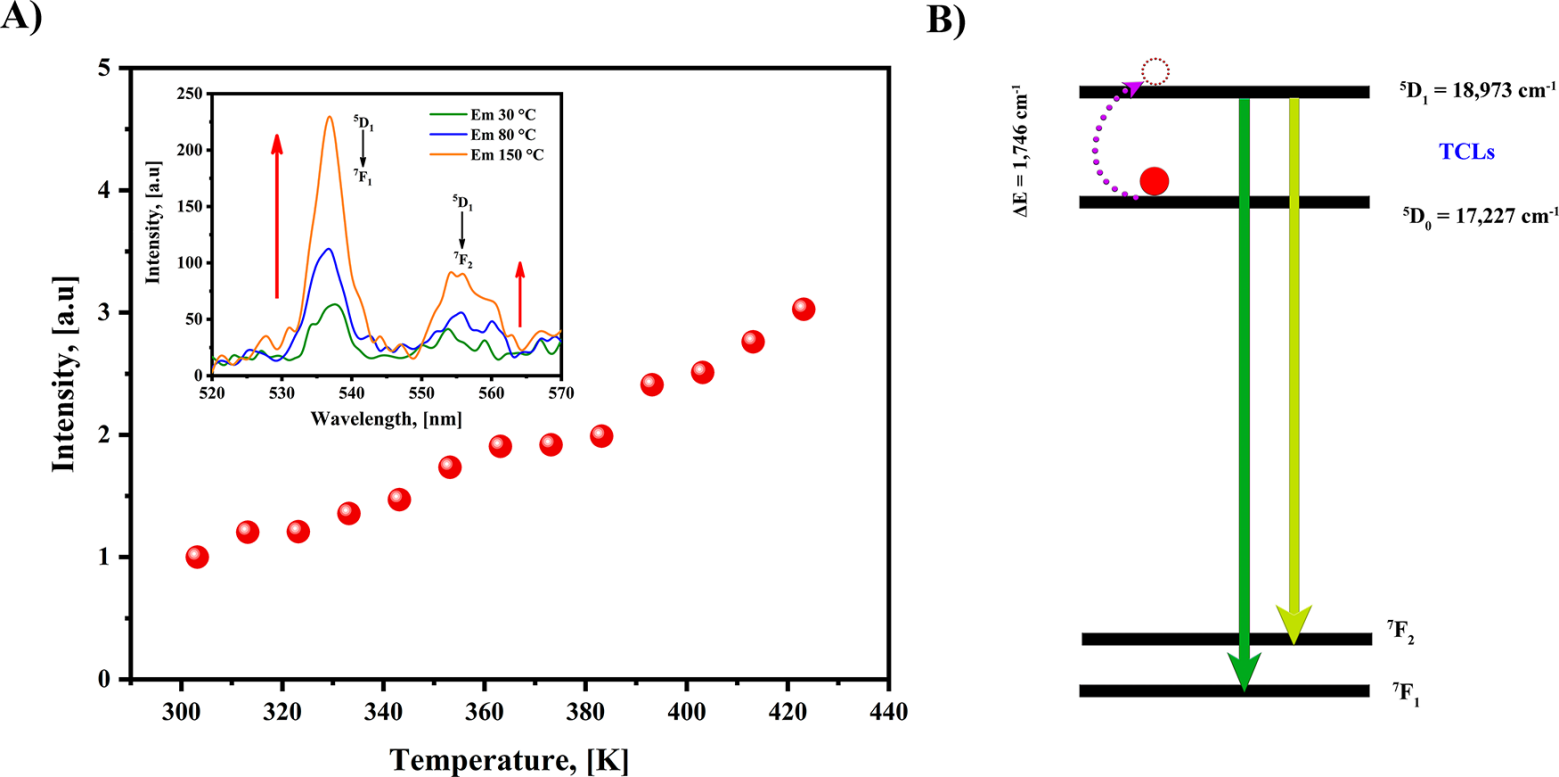
(A) Integrated normalized intensity of the ^5^D_1_ transition at λ_exc_ = 350 nm in the 30–150 °C temperature range, and (B) proposed mechanism of the thermally coupled levels.

*I*_1_ and *I*_2_ represent the intensities of ^5^D_1_ and ^5^D_0_ states, respectively; *B* and *C* are constants, Δ*E* is the energy gap between the two TCLs, *k*_B_ is the Boltzmann constant, and *T* is the absolute temperature. The energy gap between the ^5^D_1_ and ^5^D_0_ states was calculated as Δ*E* = 807 cm^–1^, close to the theoretical value of 1746 cm^–1^. The difference could be attributed to the short temperature range (30–150 °C). The plot has an *R* ^2^ of 0.97 and is presented in Figure S5. Additionally, Figure 10B displays the possible mechanism of TCL between the ^5^D_1_ and ^5^D_0_ states when the temperature increases. This effect of the TCLs could also clarify why the overall emission of the [Eu­(Phen)_2_(NO_3_)_3_] complex does not decrease significantly.

## 4 Conclusions

A europium-based red phosphor, [Eu­(Phen)_2_(NO_3_)_3_], was successfully synthesized through a simple precipitation method and completely characterized in the solid state. FTIR spectroscopy verified the coordination of two Phen ligands to the Eu^3+^ center through characteristic shifts in the ν­(C=C), ν­(C=N), δ­(C=C), and ν­(C–N) vibrational modes, while TGA analysis evidenced enhanced thermal stability of the coordinated ligands compared to free 1,10-Phenanthroline, supporting the formation of strong Eu–ligand interactions. The PXRD study, refined by the Le Bail method using the reference CCDD 252725, revealed a monoclinic structure in the *C*2/*c* space group, featuring a decacoordinated Eu^3+^ environment composed of two Phen ligands and three nitrate anions.

Furthermore, photoluminescence excitation and emission studies demonstrated that Eu^3+^ emission can be achieved through antenna effect and direct *f–f* excitation, resulting in intense red emission dominated by the (^5^D_0_ → ^7^F_1–4_) transitions. Also, the complex exhibited an outstanding photoluminescence quantum yield (Φ) of 96%, and a monoexponential luminescence decay with a lifetime (τ) of 1.096 ± 0.001 ms, indicating a single coordination environment around the Eu^3+^. Photometric analysis confirmed deep-red emission with CIE 1931 chromaticity coordinates (0.66, 0.33) and a color purity of 99%, closely matching the NTSC red standard and surpassing the chromatic performance of conventional red phosphors such as Y_2_O_3_:Eu^3+^.

Most importantly, the thermally dependent luminescence measurements revealed zero thermal quenching across the 30–150 °C interval, indicating remarkable thermal stability of luminescence. This behavior is attributed to efficient ligand-to-metal energy transfer and an appropriate energy gap between the triplet state of the Phen ligand and the ^5^D_0_ excited level of Eu^3+^, which suppresses thermally activated back energy transfer, while the presence of the thermally coupled ^5^D_0_/^5^D_1_ levels may further contribute to emission stability. Overall, these findings position the [Eu­(Phen)_2_(NO_3_)_3_] complex as a remarkably efficient, color-pure, and thermally stable red phosphor with strong potential for solid-state lighting and thermally demanding optical applications.

Compared with previously reported Eu^3+^-activated red phosphors summarized in Table 1, the [Eu­(Phen)_2_(NO_3_)_3_] compound demonstrates superior thermal robustness, particularly at 150 °C, where most inorganic systems suffer partial luminescence loss due to phonon-assisted nonradiative relaxation. In contrast, as an organic-based coordination compound, [Eu­(Phen)_2_(NO_3_)_3_] benefits from optimized triplet energy and reduced nonradiative deactivation, allowing its emission to remain stable at elevated temperatures. This distinct photophysical behavior clearly distinguishes the investigated complex from conventional inorganic Eu^3+^ phosphors and supports its novelty.

## Supplementary Material



## Notes

Thermogravimetric curves of 1,10-Phenanthroline ligand; emission spectrum focused on ^5^D_0_ → ^7^F_1,2_ at room temperature; residuals of the monoexponential luminescence decay fitting; monoclinic unit cell picture showing (*Z* = 4); and temperature-dependent integrated intensity ratio of ^5^D_0_ /^5^D_1_ transitions (PDF)
