# Supplementary material for: High-Quantum-Yield [Eu(Phen)2(NO3)3] Phosphor Exhibiting Zero Thermal Quenching under Real Operating Conditions (30–150 °C)
Source: ACS Omega. 2026 Feb 18;11(8):13989–4001. doi: 10.1021/acsomega.5c13077 (PMC12961452; doi:10.1021/acsomega.5c13077)
Supplement: Supplementary file 1 [file ao5c13077_si_001.pdf]

Supporting Information

High-Quantum-Yield [Eu(Phen)$_2$(NO$_3$)$_3$] Phosphor Exhibiting Zero Thermal Quenching Under Real Operating Conditions (30-150°C)

Karla Scanda Raymundo Silva[a*], Christian Javier Salas Juárez[b*], Raúl Erick Guzmán Silva[a], Ismael Arturo Garduño Wilches[a], Hiram Isaac Beltrán Conde[c], José Guzmán Mendoza[a]

[a]*Centro de Investigación en Ciencia Aplicada y Tecnología Avanzada, del Instituto Politécnico Nacional. Av. Legaria 694, Col. Irrigación, Miguel Hidalgo, 11500, Ciudad de México, México.*

[b]*Área de Química, Departamento de Ciencias Básicas, Universidad Autónoma Metropolitana Azcapotzalco. Av. San Pablo No. 420, Nueva el Rosario, Azcapotzalco, 02128, Ciudad de México, México.*

[c]*Área de Química de Materiales, Departamento de Ciencias Básicas, Universidad Autónoma Metropolitana Azcapotzalco, Av. San Pablo No. 420, Nueva el Rosario, Azcapotzalco, 02128, Ciudad de México, México.*

[a]Corresponding author: K. Scanda (karlascanda@gmail.com)
Ch. J. Salas Juárez (cjsj@azc.uam.mx)

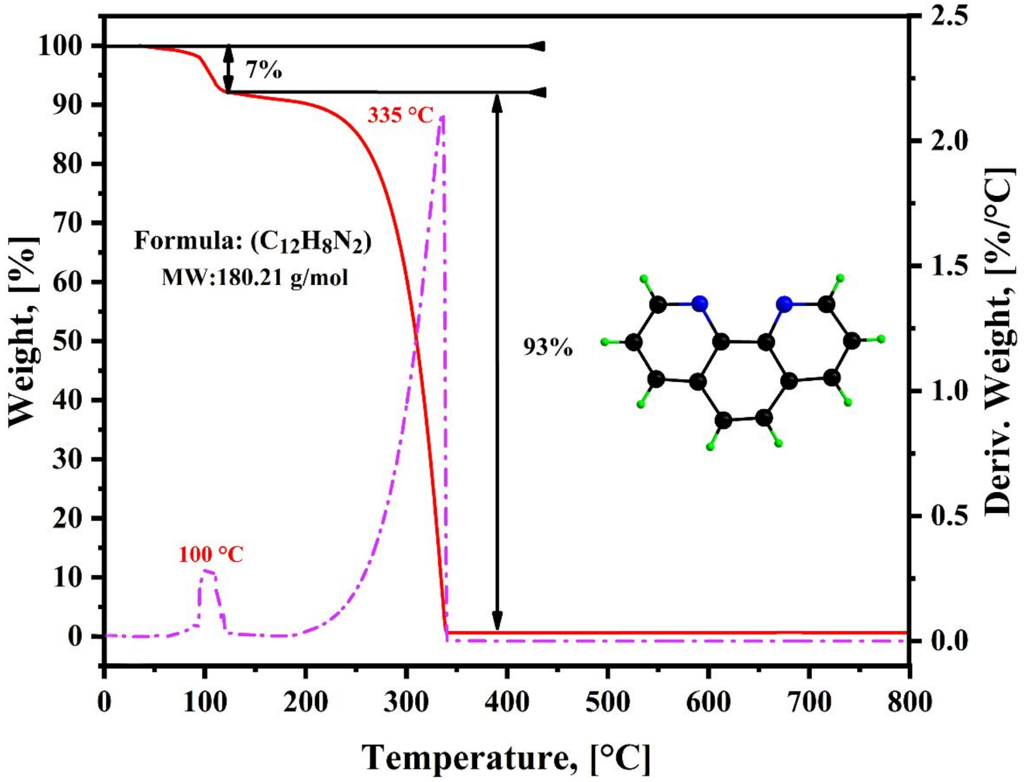

Figure S1. TGA curve and DTG curve of the 1,10-Phenanthroline ligand.

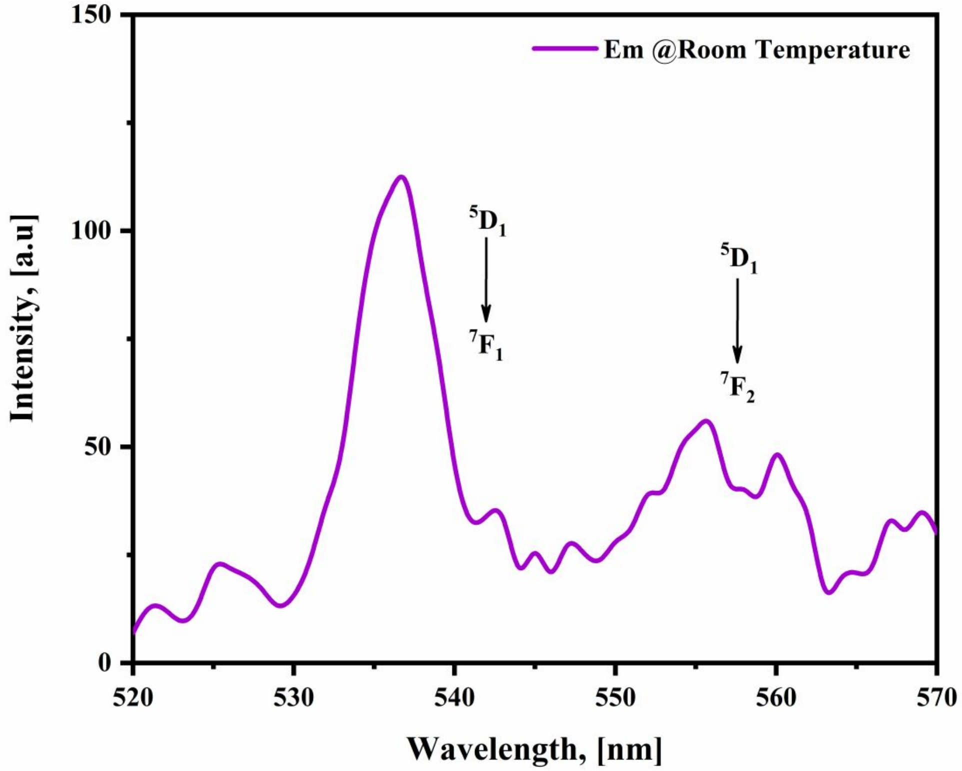

Figure S2. The emission spectrum at room temperature, λ_{exc} = 350 nm, with a zoom in the $^5D_1 \rightarrow {}^7F_{1,2}$.

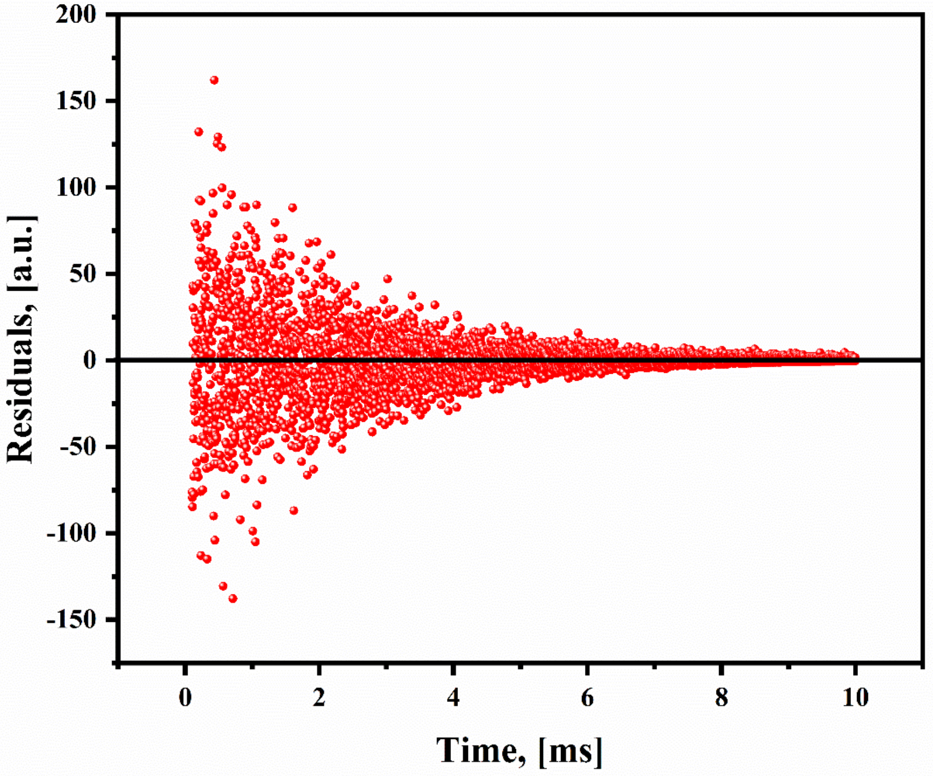

Figure S3.- The plot shows the residuals of the fit as a function of time, which are randomly distributed around zero with no systematic deviations, confirming the adequacy of the monoexponential decay model.

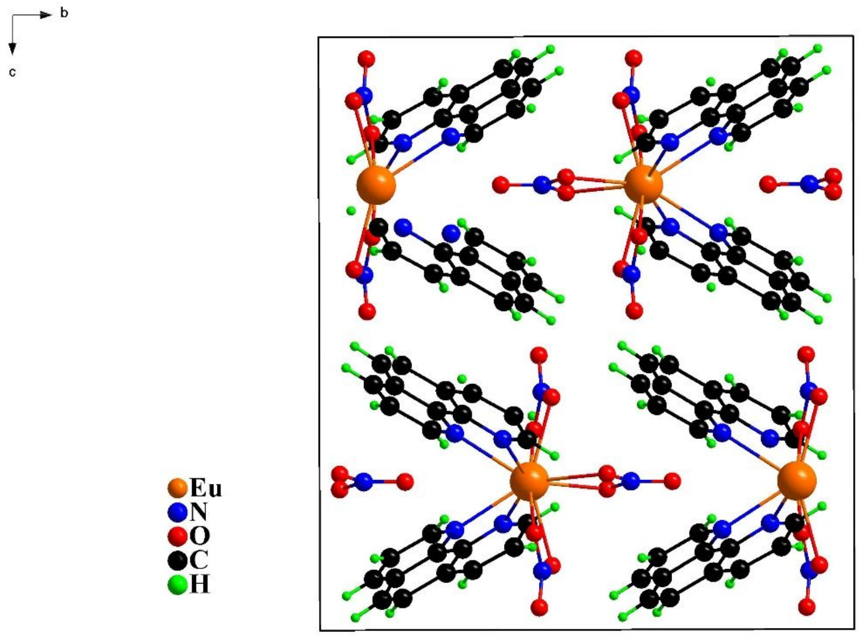

Figure S4. Representation of the monoclinic cell obtained by Le Bail refinement with four identical formula units (Z = 4).

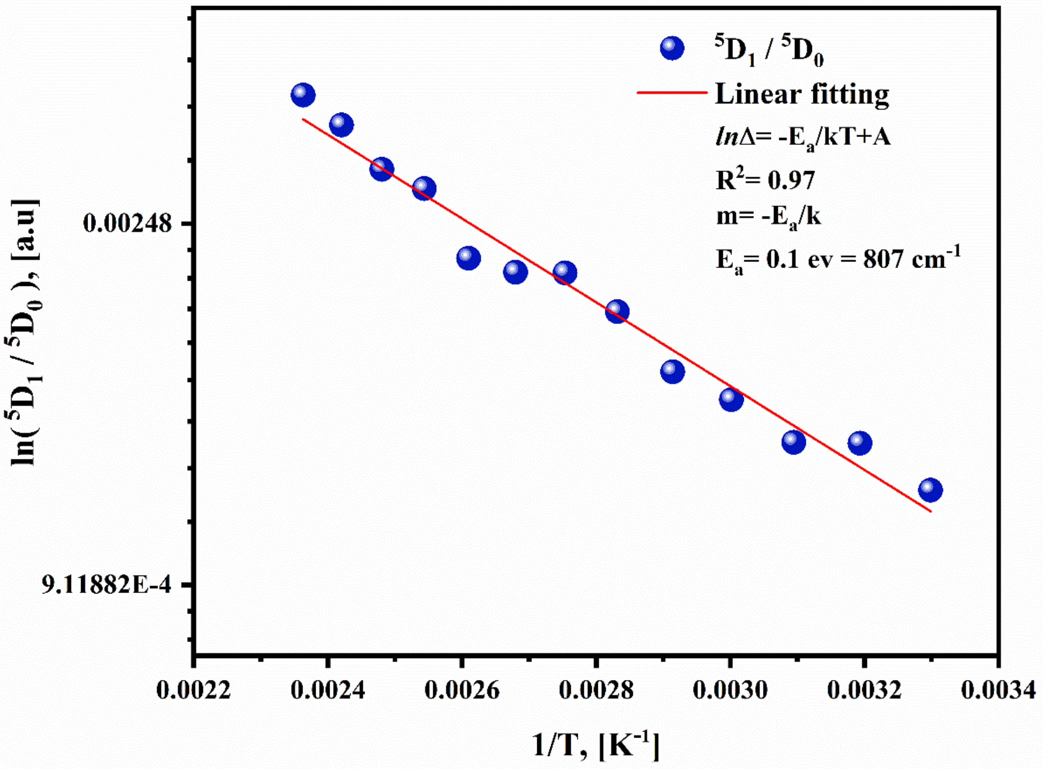

Figure S5. The temperature-dependent integrated intensity ratio of the $^5D_1/^5D_0$ transition and the linear fitting for the $[Eu(Phen)_2(NO_3)_3]$ complex.
